# Supplementary material for: Subjective Oral Health-Related Quality of Life and Objective Oral Health in People with Ectodermal Dysplasia
Source: Int J Environ Res Public Health. 2020 Dec 28;18(1):143. doi: 10.3390/ijerph18010143 (PMC7796382; doi:10.3390/ijerph18010143)
Supplement: Supplementary file 1 [file ijerph-18-00143-s001.zip › ijerph-1006532-suppl/S1.pdf]

PhOX Examination-sheet [Version 1.1]

A Self-declarations

How often have you encountered any of the following sensations in your mouth, jaw and face area in the past 30 days:

Pain:
- never
- rarely (ca. 1x per month)
- now and then (multiple per month)
- often (multiple per week)
- very often (daily)

Paresthesia: (burning, numbness, hypersensitivity, change in taste, etc.)
- never
- rarely (ca. 1x per month)
- now and then (multiple per month)
- often (multiple per week)
- very often (daily)

B Extraoral report

Pain on palpation (M. temporalis, M. masseter, mandibular joint, Gl. parotis, Gl. submandibularis)

No

Yes

Intensity: light

moderate

intense

prominence: unknown

known

C Intraoral report

Number of healthy teeth

Number of filled, crowned teeth (including implant-supported crowns)

Number of decayed teeth

Number of teeth overall (including space closure)

Number of teeth with probing-depth 3,5-5,5mm

Number of teeth with probing-depth >5,5mm

Bleeding on probing: no / yes

Number of teeth with percussion sensibility

Number of supporting zones [0-4]

Overjet (sagittal overbite)

Overbite (vertical overbite)

Maximum contact-point deviation

Lateral crossbite

Mouth opening (with dentures): straight / not straight

Maximum active mouth opening

Maximum passive mouth opening

Clefts or defects: none

Operated cleft or defect

Only in soft-tissues

Bone-defect without mouth-antrum-connection

Bone-defect with mouth-antrum-connection

Humidity: wet

partial dry

completely dry

Saliva-texture: fluid

slight foamy

no saliva

Swelling: (oral mucosa/lips/tongue)

No Yes

Area: Max. ¼ of the surface

Max. ½ of the surface

More than ½ of the surface

Whole surface

Intensity: light

intense

Color alteration: (oral mucosa/lips/tongue)

No Yes

Area: Max. ¼ of the surface

Max. ½ of the surface

More than ½ of the surface

Whole surface

Intensity: light

intense

Loss of integrity: (oral mucosa/lips/tongue)

No Yes

Area: Max. ¼ of the surface

Max. ½ of the surface

More than ½ of the surface

Whole surface

Intensity: light

intense
