# Supplementary material for: Subjective Oral Health-Related Quality of Life and Objective Oral Health in People with Ectodermal Dysplasia
Source: Int J Environ Res Public Health. 2020 Dec 28;18(1):143. doi: 10.3390/ijerph18010143 (PMC7796382; doi:10.3390/ijerph18010143)
Supplement: Supplementary file 1 [file ijerph-18-00143-s001.zip › ijerph-1006532-suppl/S2.pdf]

OHIP-G14-questionaire

	Never	Sometimes	Often	Always
Have you had trouble pronouncing any words because of probems with your teeth or mouth				
Have you felt that your sense of taste has worsened because of problems with your teeth or mouth				
Have you had painful aching in your mouth				
Have you found it uncomfortable to eat any foods because of problems with your teeth or mouth				
Have you been self-conscious because of your teeth or mouth				
Have you felt tense because of problems with your teeth or mouth				
Has been your diet been unsatisfactory because of problems with your teeth or mouth				
Have you had to interrupt meals because of problems with your teeth or mouth				
Have you found it difficult to relax because of problems with your teeth or mouth				
Have you been a bit embarressed because of problems with your teeth or mouth				
Have you been a bit irritable with other people because of problems with your teeth or mouth				
Have you had difficulty doing your usual jobs because of problems with your teeth or mouth				
Have you felt that life, in general, was less satisfying because of problems with your teeth or mouth				
Have you been totaly unable to funkction because of problems with your teeth or mouth				
